# Anxiety and depression in primary Sjögren’s syndrome: a cross-sectional study

**DOI:** 10.1186/s12888-018-1715-x

**Published:** 2018-05-16

**Authors:** Yafei Cui, Ling Xia, Lin li, Qian Zhao, Shengnan Chen, Zhifeng Gu

**Affiliations:** 1 Department of Rheumatology, Affliated Hospital of Nantong University, 20th Xisi road, 226001 Nantong, People’s Republic of China; 2 School of Nursing, Nantong University, 19th Qixiu Road, 226001 Nantong, People’s Republic of China; 3 Department of Nursing, Affliated Hospital of Nantong University, 20th Xisi road, Nantong, 226001 China

**Keywords:** Primary Sjögren’s syndrome, Depression, Anxiety, Disease activity

## Abstract

**Background:**

Prevalence of anxiety and depression is high in people with Primary Sjögren’s syndrome (pSS). However, there are currently no known reported studies about anxiety/depression in pSS patients from China. Our aim was to compare anxiety/depression in pSS patients and healthy controls; to investigate the prevalence of anxiety and/or depression among pSS patients in China; to evaluate its relationship with the disease activity, fatigue, pain, education, ocular surface disease, oral health, swallowing disorders, employment status, European League Against Rheumatism Sjögren’s Syndrome Patient Reported Index(ESSPRI) as well as to analyze potential determinants of anxiety and depression.

**Methods:**

In this study, 160 pSS patients and 170 age- and sex- matched healthy controls were included. Participants completed self-administered questionnaires, Hospital Anxiety and Depression Scale (HADS) and so on. Independent samples t-tests, χ^2^ analyses and multivariable stepwise logistic regression modeling were used to analyze the data.

**Results:**

We found 33.8% pSS patients were anxiety, and 36.9% had depression, which were significantly higher than controls. And there were significant correlations among education, employment status, disease activity, fatigue, ocular surface disease, ESSPRI, oral health, swallowing disorders and anxiety/depression. Meanwhile, logistic regression analysis revealed that oral health and swallowing disorders were significantly associated with anxiety in pSS patients; as well as fatigue was significantly associated with depression.

**Conclusions:**

The prevalence of depression and anxiety was high in adult pSS patients. Interestingly, oral health and swallowing disorders were the most important predictors of anxiety in pSS patients. Therefore, rheumatologists should pay attention to the potential mental comorbidities while managing patients with pSS and provide the basis for mental health providers in order to identify effective strategies for preventing and treating depression and anxiety among adult pSS patients. Simultaneously, rheumatologists should also focus on the oral health and swallowing disorders in pSS patients.

## Background

Primary Sjögren’s syndrome (pSS) is a systemic rheumatic autoimmune disease characterized by dryness in eyes and mouth [1]. In addition, pSS also may cause extraglandular features, with patients experiencing symptoms of pain, fatigue, anxiety, depression and negatively affect psychological, physical and social functioning [2], which has eventually the potential to impair the health-related quality of life (HR-QoL) [3]. Meanwhile, a significantly higher prevalence of mental health disorders has been consistently demonstrated in pSS patients compared with the general population [4]. As the most frequent subjective symptoms reported, depression and anxiety often have profound impacts on pSS patients’ well-being including increased incidence of cardiovascular diseases [4], associated with several psychological disorders [5], working disability [6] and may lead to disease flares. Therefore, depression and anxiety may be useful targets for interventions aimed at improving subjective health and quality of life in individuals with pSS.

Anxiety and/or depression may cause a decreased level of physical activity and decreased compliance with therapy, which can lead to worsening of disease and poorer health outcomes. pSS is a chronic debilitating condition that mainly affects the patients emotionally [7]. A common mental health problem among adults with pSS is depression. Depression was more common in patients with pSS than health controls, with a prevalence of 32-45.8% [8]. Generally depression accompanies a number of illnesses characterized by chronic inflammatory response [9]. Depression in pSS patients has been related to continuous fatigue, decreased HR-QoL, loss of work productivity, enhanced levels of physical disability and medical costs [10, 11]. Notably, anxiety was more frequent than depression in pSS. Besides, depressed pSS patients have poorer prognosis, including various complications [3]. The aim of the present study was to investigate the prevalence and characteristics of anxiety and depression in pSS and to examine the relationships between disease activity, fatigue, pain, education, the ocular surface disease, oral health, swallowing disorders, employment status, ESSPRI and anxiety/depression. Moreover, we wished to explore the possible risk factors of anxiety and depression.

## Methods

### Participants

The study was carried out in the Afliated Hospital of Nantong University from July 2016 to June 2017. pSS patients were outpatients or inpatients and healthy controls were from a population for a checkup. Of 182 patients with pSS were consecutively recruited to join in a single cross-sectional study and 160 (87.91% of the patients) participated in and finished questionnaires finally. We matched them by age and sex to avoid a possible bias. This study was approved by the Ethics Committee of the Afliated Hospital of Nantong University (2017-K003), and all subjects signed an informed consent form. Criteria for inclusion were age 18 years and older; patients conformed to the American College of Rheumatology criteria for pSS [12]. Controls were removed if they shown other systemic diseases. There was no significant demographic difference between the two groups.

### Main research tools

Participants finished self-administered questionnaires, Hospital Anxiety and Depression Scale (HADS), Fatigue Severity Scale (FSS), the Visual Analogue Scale (VAS) and so on. Additionally, the European League Against Rheumatism Sjögren’s Syndrome Disease Activity Index (ESSDAI) and Patient Reported Index (ESSPRI) were documented in patients.

### Demographics and clinical variables

Demographic features contain the following: age, sex, marital status, education level, occupation, yearly per capita income, type of medical insurance, family history of Sjögren’s syndrome, disease duration, comorbidity(autoimmune liver disease, blood system disease, cardiovascular disease, kidney disease, lung disease, thyroid disease, myositis, etc), hospitalization(admission to a hospital for treatment due to Primary Sjögren’s syndrome ever). Clinical variables of disease duration and medications use were obtained by asking patients or viewing medical records.

### Disease activity

EULAR Sjögren’s Syndrome Disease Activity Index (ESSDAI) is a clinical index assessed by rheumatologists to measure disease activity in pSS cases. There are 12 weighted fields (organ systems: cutaneous, respiratory, renal, articular, muscular, peripheral nervous system, central nervous system, haematological, glandular, constitutional, lymphadenopathy, biological), with a score ranging form 0-123 [13]. For each question, characteristics of disease activity are divided into three or four levels based on their severity [14]. Scores < 5 suggest “low disease activity”; 5 to 13 represent a “moderate disease activity”; and ≥ 14 indicate a “high disease activity” [15].

### Patient reported index

EULAR Sjögren’s Syndrome Patient Reported Index (ESSPRI) tests the intensity of symptoms and contains three domains for dryness, pain and fatigue on 0-10 numerical scales then calculating an average of the three scores [16].

### Hospital anxiety and depression scale

The Hospital Anxiety and Depression Scale (HADS) were used to measure levels of anxiety and depression, which is a self-assessment scale. Anxiety and depression were assessed respectively using the 7-item subscales, which were scored from 0 to 21 for each subscale. Scores between 0 and 7 suggest “no case”; 8 to 10 represent a “possible case”; and 11 to 21 indicate a “probable case of anxiety/depression”. Example items in the anxiety subscale include: “I can sit at ease and feel relaxed,” and “Worrying thoughts go through my mind.” Example items from the depressive subscale include: “I feel as if I am slowed down,” and “I still enjoy things I used to enjoy.” These cutoff points have been validated against clinical interviews with sensitivity and specificity approximately 0.80. Studies have reported good internal consistency for both anxiety (0.89) and depression (0.86) [17, 18].

### Other related scales

The Ocular Surface Disease Index (OSDI) [19] assesses symptoms of ocular irritation consistent with dry eye disease and their impact on vision-related function. The total OSDI score ranges from 0 to 100, with higher scores indicating more severe symptoms. And it has been validated in Chinese populations [20]; the M.D. Anderson Dysphagia Inventory (MDADI) [21] measures patient’s swallowing disorders and the Oral Health Impact Profile 14 (OHIP-14) [22] was used to survey oral distress(score ranges from 0 to 100, with higher scores indicating more severe symptoms).

### Data analysis

Data were analyzed using the computer program, SPSS 21. The data were shown as means ± SD for continuous data and as frequencies (%) for categorical data. Variables were used univariate tests (independent samples t-tests and χ^2^ tests) of the group difference. Correlation analysis was used for exploration of the relation between two variables. All variables with a significant association with anxiety/depression were entered into a multiple stepwise logistic regression model with the dichotomous anxiety/depression as the dependent variable, to explore the predictors of psychological status. *P* < 0.05(two-tail) was considered statistically significant.

## Results

### Patient features

22 pSS patients and 16 controls didn’t complete the questionnaires, resulting in the admission of 160 pSS patients and 170 healthy subjects with matched age and sex in this study. The mean age of pSS patients was 50.54 ± 12.22 years and the mean disease duration was 4.5 years. Demographic, clinical, and laboratory characteristics of pSS patients and controls are shown in Table 1. Anti-SSA antibody and anti-SSB antibody were positive in 85 (81%) and 53 (50.5%), respectively. 55 outpatients did not perform the anti-SSA antibody and antiSSB antibody examination and 57 outpatients did not perform the RF examination. 114 (71.3%) pSS patients showed moderate to high disease activity (ESSDAI ≥5). The score of depression is higher in pSS patients than controls(6.71 ± 4.32 vs 3.87 ± 2.80). And the score of anxiety is higher in pSS patients than controls(6.52 ± 4.06 vs 3.02 ± 2.78). There was no significant difference in the age, sex, marital status, education level, occupation, income/year between the two groups (*P* > 0.05).

**Table 1:** summarizes the demographic and clinical characteristics of the participants in the study

Clinical characteristics	pSS(*n* = 160)	Controls(*n* = 170)	*t/x* ^*2*^	*p*
Age, mean ± sd years	51.09 ± 12.11	50.14 ± 11.22	0.32	0.446
Gender, female, no. (%)	152(95)	162(95.3)	0.02	0.901
Marital status, no. (%)			3.50	0.061
Married	151 (94.4)	167 (98.2)		
Other marital status	9 (5.6)	3 (1.8)		
Education, years	12.19 ± 4.33	12.03 ± 4.62	0.33	0.740
Employment status, no. (%)			1.28	0.259
Employed	71(44.4)	86(50.6)		
Unemployed	89(55.6)	84(49.4)		
Income/person/year, yuan, no. (%)			1.65	0.569
< 15,000 yuan	55 (34.37)	49(28.8)		
15,000-33,000 yuan	49 (30.63)	62(36.5)		
≥ 33,000 yuan	56(35)	59(34.7)		
Type of medical insurance, n (%)			4.33	0.115
With basic medical insurance	146(91.3)	164(96.5)		
Self-pay	13(8.1)	6(3.5)		
Other	1(0.6)	0(0)		
HADS-A, mean ± sd	6.52 ± 4.06	3.02 ± 2.78	9.08	0.000
HADS-D, mean ± sd	6.71 ± 4.32	3.87 ± 2.80	7.05	0.000
Disease duration, mean ± sd years	4.498 ± 5.44	–		–
OSDI, mean ± sd	29.14 ± 25.12	–		–
VAS pain (range 0–10), mean ± sd	2.36 ± 2.65	–		–
ESR, mean ± sd mm/h	30.65 ± 24.13	–		–
RF positivity, yes, no. (%)	51(49.5)	–		–
Hospitalization, yes, n (%)	108(67.5)	–		–
Family history, yes, n (%)	23(14.4)	–		–
Comorbid conditions, yes, n (%)	102(63.75)	–		–
ESSDAI	9.6 ± 7.3	–		–
ESSPRI	4.53 ± 1.95	–		–
FSS	3.89 ± 1.59	1.64 ± 0.89	15.76	0.000
OHIP-14	12.89 ± 11.38	3.41 ± 4.92	9.29	0.000
MDADI	72.33 ± 18.83	–		–
IgG (g/L)	18.93 ± 6.65	–		–
C3 (g/L)	0.81 ± 0.27	–		–
C4 (g/L)	0.18 ± 0.65	–		–
ANA ≥1: 320	96(91.4)	–		–
Anti-SSA ≥ 200 RU/mL	85(81)	–		–
Anti-SSB ≥ 20 RU/mL	53(50.5)	–		–

*VAS* visual analog scale, *ESR* erythrocyte sedimentation rate, *RF* rheumatoid factor, *HADS* hospital anxiety and depression scale, *HADS-A* hospital anxiety scale, *HADS-D* hospital depression scale, *ESSDAI* EULAR Sjögren’s syndrome disease activity index, *ANA* antinuclear antibodies, *IgG* Immunoglobulin G, *C3* complement C3, *C4* complement C4, *OSDI* the ocular surface disease index

### Differences between anxiety/depression and non-anxiety/depression patients

As indicated in Table 2, demographic, clinical and psychological variables were compared between anxiety/depression and non-anxiety/depression pSS patients. Depressed pSS patients had more serious disease activity, higher ESR scores and unemployment rate, lower education level, severer total pain, compared with non-depression patients (*p* < 0.05). However, no similar results were found in anxiety. For age, gender, marital status, disease duration, type of medical insurance, complication, hospitalization and family history, no statistically significant difference was found between the two groups (*P*>0.05). Enhanced pain, physical limitations, and fatigue may be interpreted as increased activity of these diseases.

**Table 2:** Differences between different factors of anxiety/depression and non-anxiety/depression patients

*Clinical characteristics*	non-anxiety	Anxiety	*p*	non-depression	depression	*p*
Age, mean ± sd years^a^	50.08 ± 11.90	51.44 ± 12.90	0.508	49.69 ± 11.96	51.12 ± 12.68	0.228
Gender, female, no. (%)^b^	100(94.3)	55 (96.3)	0.878	97(96)	55 (93.2)	0.679
Marital status, no. (%)^b^			1.000			0.401
Married	100 (94.3)	51 (94.4)		97 (96)	54 (91.5)	
Other marital status	6 (5.7)	3 (5.6)		4 (4)	5 (8.5)	
Education, years, no. (%)^a^	12.43 ± 4.42	11.72 ± 4.16	0.327	13.00 ± 3.96	10.81 ± 4.62	0.002^*^
Employment status, no. (%)^b^			0.064			0.049^*^
Employed	53 (50)	18 (33.3)		51 (50.5)	20 (33.9)	
Unemployed	53 (50)	36 (66.7)		50 (49.5)	39 (66.1)	
Income/person/year, yuan, no. (%)^b^			0.423			0.094
< 15,000 yuan	34 (32.1)	21 (38.9)		31 (30.7)	24 (40.7)	
15,000-33,000 yuan	36 (34)	13 (24.1)		37 (36.6)	12 (20.3)	
≥ 33,000 yuan	36 (34)	20 (37)		33 (32.7)	23 (39)	
Type of medical insurance, n (%)^b^			0.749			0.660
With basic medical insurance	96 (90.6)	50 (92.6)		91 (90.1)	55 (93.2)	
Self-pay	9 (8.5)	4 (7.4)		9 (8.9)	4 (6.8)	
Other	1 (0.9)	0 (0)		1 (1)	0 (0)	
Disease duration, mean ± sd years^a^	4.48 ± 5.61	4.53 ± 5.15	0.957	4.30 ± 4.72	4.83 ± 6.53	0.557
VAS pain (range 0–10), mean ± sd^a^	2.12 ± 2.51	2.83 ± 2.98	0.127	2.35 ± 1.95	3.07 ± 2.99	0.016^*^
ESR, mean ± sd mm/h^a^	28.10 ± 21.38	35.87 ± 28.47	0.085	27.10 ± 20.04	36.78 ± 29.10	0.027^*^
OSDI, mean ± sd^a^	25.07 ± 22.40	37.15 ± 28.28	0.008^*^	23.82 ± 20.81	38.26 ± 29.14	0.001^*^
RF positivity, yes, no. (%)^b^	29 (46)	22 (55)	0.422	30 (49.2)	21 (50)	1.000
Hospitalization, yes, n (%)^b^	68 (64.2)	40 (74.1)	0.218	68 (67.3)	40 (67.8)	1.000
Family history, yes, n (%)^b^	15 (14.2)	8 (14.8)	1.000	17 (16.8)	6 (10.2)	0.351
Comorbid conditions, yes, n (%)^b^	66 (62.3)	36 (66.7)	0.607	61 (60.4)	41 (69.5)	0.307
ESSDAI^a^	9.16 ± 7.73	10.48 ± 6.37	0.281	8.73 ± 7.35	11.10 ± 7.03	0.047^*^
IgG (g/L)^a^	19.05 ± 6.72	18.72 ± 6.60	0.806	18.99 ± 6.54	18.84 ± 6.85	0.909
C3 (g/L)^a^	0.80 ± 0.26	0.82 ± 0.28	0.781	0.80 ± 0.24	0.82 ± 0.30	0.632
C4 (g/L)^a^	0.18 ± 0.07	0.17 ± 0.06	0.627	0.18 ± 0.66	0.17 ± 0.06	0.552
ANA ≥1: 320^b^	61 (91)	35 (92.1)	1.000	60 (93.8)	36 (87.8)	0.481
Anti-SSA ≥ 200 RU/mL^b^	54 (80.6)	31 (81.6)	1.000	50 (78.1)	35 (85.4)	0.449
Anti-SSB ≥ 20 RU/mL^b^	32 (47.8)	21 (55.3)	0.544	29 (45.3)	24 (58.5)	0.231

*VAS* visual analog scale, *ESR* erythrocyte sedimentation rate, *RF* rheumatoid factor, *HADS* hospital anxiety and depression scale, *ESSDAI* EULAR Sjögren’s syndrome disease activity index, *ANA* antinuclear antibodies, *IgG* immunoglobulin G, *C3* complement C3, *C4* complement C4, *OSDI* the ocular surface disease index.^*^ = *p*<0.05; ^a^ = t-tests; ^b^ = χ^2^ analyses

### Correlations among fatigue, disease activity and the HADS-depression/anxiety in pSS patients

As shown in Table 3, we found there were significant correlations among pain, employment status, fatigue, the ocular surface disease, swallowing disorders and oral health were significantly associated with the HADS-depression (*p* < 0.05). In addition, fatigue, disease activity, ESSPRI, employment status, the ocular surface disease, swallowing disorders and oral health were significantly associated with the HADS-anxiety (*p* < 0.05).

**Table 3:** Correlations among fatigue, pain, education etc. and the HADS-depression/anxiety in pSS patients

*correlative variable*	Anxiety		depression	
	r	*p*	r	*p*
OHIP	−0.353	0.000^**^	−0.228	0.004^**^
OSDI	−0.228	0.004^**^	− 0.278	0.000^**^
FSS	−0.215	0.006^**^	−0.358	0.000^**^
Pain	−0.127	0.109	−0.204	0.010^**^
ESSDAI	−0.086	0.281	−0.157	0.047^*^
ESSPRI	− 0.169	0.053	−0.268	0.002^**^
Education	0.078	0.327	0.244	0.002^**^
Employment status	0.159	0.045^*^	0.161	0.042^*^
Swallowing disorders	0.398	0.000^**^	0.233	0.003^**^

^*^p<0.05;^* *^ *p*<0.01

### Logistic regression analysis for the HADS-anxiety and depression

We used logistic regression analysis to investigate predictors of anxiety and depression, the result revealed oral health(odds ratio = 0.956; *P*<0.05) and swallowing disorders(odds ratio = 1.036; *P*<0.05) were significantly associated with the HADS-anxiety in pSS patients, which means oral health and swallowing disorders were the predictors of anxiety in pSS patients(Table 4). Meanwhile, fatigue(odds ratio = 0.587; *P*<0.05) was significantly accounted for the HADS-depression (Table 5).

**Table 4:** Result of analysis of forward stepwise ordered logit regression models in anxiety

*Predictors*	B	S.E.	*P*	OR (95%CI)
OHIP	−0.045	0.019	0.017	0.956(0.921, 0.992)
Swallowing disorders	0.035	0.011	0.002	1.036(1.013, 1.059)

*CI* confdence interval, *OHIP* oral health impact profile

**Table 5:** Result of analysis of forward stepwise ordered logit regression models in depression

*Predictors*	β	S.E.	*P*	OR (95%CI)
FSS	−0.533	0.123	0.000	0.587 (0.461, 0.747)

*CI* confdence interval, *FSS* Fatigue severity scale

## Discussion

According to this study, the prevalence of depression and anxiety is 36.9%/33.8% among people with pSS in China and Chinese pSS patients are more likely to suffer from depression and anxiety than healthy people. As far as we know, this is a study chiefly researching the relations between disease activity, fatigue, pain, education, ocular surface disease, oral health, swallowing disorders, employment status, ESSPRI and anxiety/depression in Chinese pSS patients.

Depression patients had higher disease activity scores, higher level of ESR, higher unemployment rate, lower education level, severer total pain, compared with depression patients. One explanation would be that pSS and pain can cause adverse coping ways, work restriction and impaired quality of life, which might in turn bring about a further depressive flare. However, no similar results were found in anxiety. A recent meta-analysis showed that prevalence estimates for depression in pSS range between 8.33 and 75.56% [23]. As reported previously, depression in pSS patients is associated with poorer health status, including low quality of life [24], suicide risk [25], increased medical costs [26], lower rate of treatment compliance [27], and even increased mortality. Similarly, Lendrem et al. [28] found that pSS patients complaining any problem in mobility, self-care, daily activities, pain/malaise, and anxiety/depression were higher than normal people. Moreover, Shen et al. [29] reported that pSS may increase the risk of ensuing newly diagnosed depressive disorder, anxiety disorder that might damage quality of life. Depression was found more common in pSS patients than controls, with a prevalence of 38.3% [30], which is consistent with our findings. However, some other researches made inconsistent conclusions, and need further discussions.

We could not detect a difference in sex because of the small male proportion in this study. There are disputed data whether active disease adds the susceptibility to anxiety/depression. However, we found a positive relationship between disease activity and anxiety/depression. Pain severity were also related to an increased risk of anxiety and depression. This finding supports the opinion of a mutually reinforcing mechanism between pain and depression and indicates the importance of clarifying neurobiological links for the sake of optimizing pain and depression management. pSS affects patients’ HR-QoL, mental status and relationships with family [31]. Fatigue is significantly associated with various psychological factors, including depression [32], anxiety [33] in pSS patients.

Patients with pSS usually have characteristic chronic symptoms of ocular and oral dryness. While the pathomechanism of pSS is still unclear, environmental factors are considered to trigger abnormal immunological reactions in people with genetic susceptibility [34]. Ocular and oral dryness were the most frequently symptoms in pSS patients reported by 44 and 39%, respectively [11]. While corticosteroid and immunomodulatory drugs are often ineffective in the treatment of oral and ocular symptoms of pSS. The possible explanation is that manifestations of oral and ocular dryness are vague in the early phase of the disease and usually ignored by patients and clinicians alike [35]. The pSS patients in our study had severer ocular expressions and signs of dry eye and reported severer anxiety and depression. Furthermore, studies show that pSS patients have a decreased salivary flow with high risk of dental caries, enhanced susceptibility to oral candidiasis, tooth loss, severer periodontal destruction, pain during chewing, pararthria, tongue palpitation and angular cheilitis [36–38]. Anxious pSS individuals, because of their avoidant behaviours, often have poorer dental health. Oral dryness in pSS may impair functions such as chewing, talking or swallowing, and lead to a “choking” sensation, which might cause anxiety [39]. Fortunately, Hydroxychloroquine has shown improvement of subjective oral symptoms in patients with pSS. Therefore, it is essential that patients with pSS undergo regular oral examinations and medication therapy.

The amount of saliva in pSS patients was significantly less than controls, and these patients perceived their swallowing to be impaired [40]. Moreover, dysphagia in pSS patients increase with disease severity and anxiety/depression, indicating the important for increased recognition and management of dysphagia. Since eating and drinking form an important part of social interaction, dysphagic pSS patients often eat in seclusion because of shame. And they worry choking on their food or develop aspiration pneumonia. Frequent worries can decrease quality of life even more and aggravate the anxiety. Hence, it is important to strengthen the assessment of the swallowing function of the pSS patient. Educational programs might help an early identification of the disease and lessen the emotional burden of patients during the initial course of disease [41]. In addition, lacking of knowledge about pSS may lead to anxiety/depression, so patient education about pSS may relieve anxiety and depression.

The large sample size available for this analysis and systematic investigation of the disease activity in pSS patients were major strengths for this study. However, there are some limitations. Firstly, this study is a single-center research. Secondly, the data analysed were cross-sectional, and thus we were unable to comment on causal relationships. Thirdly, some of the positive findings cannot survive correction for multiple comparisons. Additionally, prospective studies on pSS patients’ anxiety and depression should be conducted to support the development of effective interventions to manage their anxiety and depression in the future.

## Conclusion

Patients with pSS complained more commonly of anxiety and depression than controls, and oral health and swallowing disorders were greatly relevant to anxiety in pSS patients. Clinicians should focus on the potential mental comorbidities while managing patients with pSS. Simultaneously, rheumatologists should also pay more attention to the oral health and swallowing disorders in pSS patients. Early identification and proper intervention are therefore vital to minimize the negative effect of anxiety and depression on the patient’s QoL and outcome of their disease. Accordingly, the findings in our study can be used for prevention and intervention of psychological distress(anxiety and depression) among pSS patients. The findings have significant policy or practical implications. Finally, further studies are indispensable to search potential pathophysiologic pathways that might interpret anxiety and depression in pSS.

## Abbreviations

ESSDAI: European League Against Rheumatism Sjögren’s Syndrome Disease Activity Index
ESSPRI: European League Against Rheumatism Sjögren’s Syndrome Patient Reported Index
FSS: Fatigue severity scale
HADS: Hospital Anxiety and Depression Scale
HR-QoL: Health-related quality of life
MDADI: the M.D. Anderson Dysphagia Inventory
OHIP-14: the Oral Health Impact Profile 14
OSDI: the Ocular Surface Disease Index
pSS: Primary Sjögren’s syndrome
VAS: the Visual Analogue Scale

## References

[1] Karlsen M, Jakobsen K, Jonsson R, Hammenfors D, Hansen T, Appel S (2017). Expression of toll-like receptors in peripheral blood mononuclear cells of patients with primary Sjögren's syndrome. Scand J Immunol.

[2] Koh JH, Kwok SK, Lee J (2017). Pain, xerostomia, and younger age are major determinants of fatigue in Korean patients with primary Sjogren's syndrome: a cohort study. Scand J Rheumatol.

[3] Kotsis K, Voulgari PV, Tsifetaki N, Drosos AA, Carvalho AF, Hyphantis T (2014). Illness perceptions and psychological distress associated with physical health-related quality of life in primary Sjögren’s syndrome compared to systemic lupus erythematosus and rheumatoid arthritis. Rheumatol Int.

[4] Anyfanti P, Pyrpasopoulou A, Triantafyllou A (2014). Association between mental health disorders and sexual dysfunction in patients suffering from rheumatic diseases. J Sex Med.

[5] Lendrem D, Mitchell S, McMeekin P (2014). Health-related utility values of patients with primary Sjogren's syndrome and its predictors. Ann Rheum Dis.

[6] Westhoff G, Dorner T, Zink A (2012). Fatigue and depression predict physician visits and work disability in women with primary Sjogren's syndrome: results from a cohort study. Rheumatology.

[7] Van LN, Bossema ER, Van MH (2012). Dealing with emotions when the ability to cry is hampered: emotion processing and regulation in patients with primary Sjögren's syndrome. Clin Exp Rheumatol.

[8] Koçer B, Tezcan ME, Batur HZ (2016). Cognition, depression, fatigue, and quality of life in primary Sjögren's syndrome: correlations. Brain Behav.

[9] Buras A, Waszkiewicz N, Szulc A, Sierakowski S (2012). Monocytic parameters in patients with rheumatologic diseases reflect intensity of depressive disorder. Pol Merkur Lekarski.

[10] Inal V, Kitapcioglu G, Karabulut G, Keser G, Kabasakal Y (2010). Evaluation of quality of life in relation to anxiety and depression in primary Sjogren's syndrome. Mod Rheumatol.

[11] Segal B, Bowman SJ, Fox PC (2009). Primary Sjogren's syndrome: health experiences and predictors of health quality among patients in the United States. Health Qual Life Out.

[12] Shiboski SC, Shiboski CH, Criswell L (2012). American College of Rheumatology classification criteria for Sjögren’s syndrome: a data-driven, expert consensus approach in the Sjögren's international collaborative clinical alliance cohort. Arthritis Care Res.

[13] Seror R, Bowman SJ, Brito-Zeron P (2015). EULAR Sjogren's syndrome disease activity index (ESSDAI): a user guide. RMD Open.

[14] Seror R, Ravaud P, Bowman SJ (2010). EULAR Sjogren's syndrome disease activity index: development of a consensus systemic disease activity index for primary Sjogren's syndrome. Ann Rheum Dis.

[15] Seror R, Bootsma H, Saraux A (2016). Defining disease activity states and clinically meaningful improvement in primary Sjögren's syndrome with EULAR primary Sjögren's syndrome disease activity (ESSDAI) and patient-reported indexes (ESSPRI). Ann Rheum Dis.

[16] Seror R, Ravaud P, Mariette X (2011). EULAR Sjogren’s syndrome patient reported index (ESSPRI): development of a consensus patient index for primary Sjogren's syndrome. Ann Rheum Dis.

[17] Cordingley L, Prajapati R, Plant D (2014). Impact of psychological factors on subjective disease activity assessments in patients with severe rheumatoid arthritis. Arthrit Care Res.

[18] Olsson I, Mykletun A, Dahl AA (2005). The hospital anxiety and depression rating scale: a cross-sectional study of psychometrics and case finding abilities in general practice. BMC Psychiatry.

[19] Schiffman RM, Christianson MD, Jacobsen G, Hirsch JD, Reis BL (2000). Reliability and validity of the ocular surface disease index. Arch Ophthalmol.

[20] Zhang Y, Lin T, Jiang A, Zhao N, Gong L (2016). Vision-related quality of life and psychological status in Chinese women with Sjogren's syndrome dry eye: a case-control study. BMC Womens Health.

[21] Pierce JL, Tanner K, Merrill RM, Miller KL, Kendall KA, Roy N (2016). Swallowing disorders in Sjögren's syndrome: prevalence, risk factors, and effects on quality of life. Dysphagia.

[22] Enger TB, Palm Ø, Garen T, Sandvik L, Jensen JL (2011). Oral distress in primary Sjögren’s syndrome: implications for health-related quality of life. Eur J Oral Sci.

[23] Cui Y, Li L, Yin R (2017). Depression in primary Sjögren’s syndrome: a systematic review and metaanalysis. Psychol Health Med.

[24] Morgane M, Cornec D, Chastaing M (2016). Sicca symptoms are associated with similar fatigue, anxiety, depression, and quality-of-life impairments in patients with and without primary Sjögren’s syndrome. Joint Bone Spine.

[25] Choo C, Diederich J, Song I, Ho R (2014). Cluster analysis reveals risk factors for repeated suicide attempts in a multi-ethnic Asian population. Asian J Psychiatr.

[26] Ho RC, Mak K, Chua AN, Ho CS, Mak A (2014). The effect of severity of depressive disorder on economic burden in a university hospital in Singapore. Expert Rev Pharm Out.

[27] Jamilloux Y, Sarabi M, Kerever S (2015). Adherence to online monitoring of patient-reported outcomes by patients with chronic inflammatory diseases: a feasibility study. Lupus.

[28] Lendrem D, Mitchell S, McMeekin P (2014). Health-related utility values of patients with primary Sjögren's syndrome and its predictors. Ann Rheum Dis.

[29] Shen CC, Yang AC, Kuo BI (2015). Risk of psychiatric disorders following primary Sjögren syndrome: a Nationwide population-based retrospective cohort study. J Rheumatol.

[30] Morreale M, Marchione P, Giacomini P (2014). Neurological involvement in primary Sjogren syndrome: a focus on central nervous system. PLoS One.

[31] Karageorgas T, Fragioudaki S, Nezos A, Karaiskos D, Moutsopoulos HM, Mavragani CP (2016). Fatigue in primary Sjögren's syndrome: clinical, laboratory, psychometric, and biologic associations. Arthrit Care Res..

[32] Ibn Yacoub Y, Rostom S, Laatiris A, Hajjaj-Hassouni N (2012). Primary Sjögren’s syndrome in Moroccan patients: characteristics, fatigue and quality of life. Rheumatol Int.

[33] Milin M, Cornec D, Chastaing M (2016). Sicca symptoms are associated with similar fatigue, anxiety, depression, and quality-of-life impairments in patients with and without primary Sjögren's syndrome. Joint Bone Spine.

[34] KANG JH, LIN HC (2010). Comorbidities in patients with primary sjogren's syndrome: a registry-based case-control study. J Rheumatol.

[35] Leung KCM, McMillan AS, Wong MCM, Leung WK, Mok MY, Lau CS (2008). The efficacy of cevimeline hydrochloride in the treatment of xerostomia in Sjogren's syndrome in southern Chinese patients: a randomised double-blind, placebo-controlled crossover study. Clin Rheumatol.

[36] Ambrosio LM, Rovai ES, Franca BN (2017). Effects of periodontal treatment on primary sjogren's syndrome symptoms. Braz Oral Res.

[37] Lu M, Jheng C, Tsai T, Koo M, Lai N (2014). Increased dental visits in patients prior to diagnosis of primary Sjögren's syndrome: a population-based study in Taiwan. Rheumatol Int.

[38] Olate S, Munoz D, Neumann S, Pozzer L, Cavalieri-Pereira L, de Moraes M (2014). A descriptive study of the oral status in subjects with Sjogren's syndrome. Int J Clin Exp Med.

[39] Rostron J, Rogers S, Longman L (2002). Health-related quality of life in patients with primary Sjögren's syndrome and xerostomia: a comparative study. Gerodontology.

[40] Roguspulia NM, Logemann JA (2011). Effects of reduced saliva production on swallowing in patients with Sjögren's syndrome. Dysphagia.

[41] Lackner A, Ficjan A, Stradner MH (2017). It’s more than dryness and fatigue: the patient perspective on health-related quality of life in primary Sjögren's syndrome - a qualitative study. PLoS One.

